# Supplementary material for: Troponin Cut-Offs for Acute Myocardial Infarction in Patients with Impaired Renal Function—A Systematic Review and Meta-Analysis
Source: Diagnostics (Basel). 2022 Jan 21;12(2):276. doi: 10.3390/diagnostics12020276 (PMC8871519; doi:10.3390/diagnostics12020276)
Supplement: Supplementary file 1 [file diagnostics-12-00276-s001.zip › diagnostics-1540725-supplementary.pdf]

Search Strategy

EMBASE (21.01.2020, 1841 hits)

Exp Troponin/ OR (Troponin* OR Troponin Complex* OR TnT OR TnI OR Cardiac troponin* OR cTroponin* OR cTnT OR cTnI OR High sensitive troponin* OR Hs-TnT OR Hs-TnI OR Hs-Troponin* OR Hs-cTn*)

AND

Exp Chronic Kidney Failure/ OR (Chronic Renal Insufficienc* OR Chronic Kidney Insufficienc* OR Chronic Kidney Disease* OR CKD OR Chronic Renal Disease* OR Chronic Kidney Failure* OR Chronic Renal Failure* OR End-stage Kidney Disease* OR End Stage Kidney Disease* OR End-Stage Renal Disease* OR End Stage Renal Disease* OR ESRD OR Chronic Kidney Disorder* OR Chronic Nephropathy OR (Chronic Kidney Disease-mineral and bone disorder*) OR CKD-MBD OR (CKD-mineral and bone disorder*))

Embase Classic+Embase 1947 to 2020 January 20

Enter keyword or phrase (* or $ for truncation) Keyword Author Title Journal Search

Limits (expand) Include Multimedia Map Term to Subject Heading

Search History (7) View Saved

#	Searches	Results	Type	Actions	Annotations
1	exp troponin/	57504	Advanced	Display Results | More	
2	(Troponin* or Troponin Complex* or TnT or Tnl or Cardiac troponin* or cTroponin* or cTnT or cTnl or High sensitive troponin* or Hs-TnT or Hs-Tnl or Hs-Troponin* or Hs-cTn*).mp. [mp=title, abstract, heading word, drug trade name, original title, device manufacturer, drug manufacturer, device trade name, keyword, floating subheading word, candidate term word]	66708	Advanced	Display Results | More	
3	1 or 2	66708	Advanced	Display Results | More	
4	exp chronic kidney failure/	96135	Advanced	Display Results | More	
5	(Chronic Renal Insufficienc* or Chronic Kidney Insufficienc* or Chronic Kidney Disease* or CKD or Chronic Renal Disease* or Chronic Kidney Failure* or Chronic Renal Failure* or End-stage Kidney Disease* or End Stage Kidney Disease* or End-Stage Renal Disease* or End Stage Renal Disease* or ESRD or Chronic Kidney Disorder* or Chronic Nephropathy or (Chronic Kidney Disease-mineral and bone disorder*) or CKD-MBD or (CKD-mineral and bone disorder*)).mp. [mp=title, abstract, heading word, drug trade name, original title, device manufacturer, drug manufacturer, device trade name, keyword, floating subheading word, candidate term word]	224356	Advanced	Display Results | More	
6	4 or 5	226800	Advanced	Display Results | More	
7	3 and 6	1841	Advanced	Display Results | More	

MEDLINE (21.01.2020, 694)

Exp Troponin/ OR (Troponin* OR Troponin Complex* OR TnT OR TnI OR Cardiac troponin* OR cTroponin* OR cTnT OR cTnI OR High sensitive troponin* OR Hs-TnT OR Hs-TnI OR Hs-Troponin* OR Hs-cTn*)

AND

Exp Chronic Renal Insufficiency/ OR (Chronic Renal Insufficienc* OR Chronic Kidney Insufficienc* OR Chronic Kidney Disease* OR CKD OR Chronic Renal Disease* OR Chronic Kidney Failure* OR Chronic Renal Failure* OR End-stage Kidney Disease* OR End Stage Kidney Disease* OR End-Stage Renal Disease* OR End Stage Renal Disease* OR ESRD OR Chronic Kidney Disorder* OR Chronic Nephropathy OR (Chronic Kidney Disease-mineral and bone disorder*) OR CKD-MBD OR (CKD-mineral and bone disorder*))

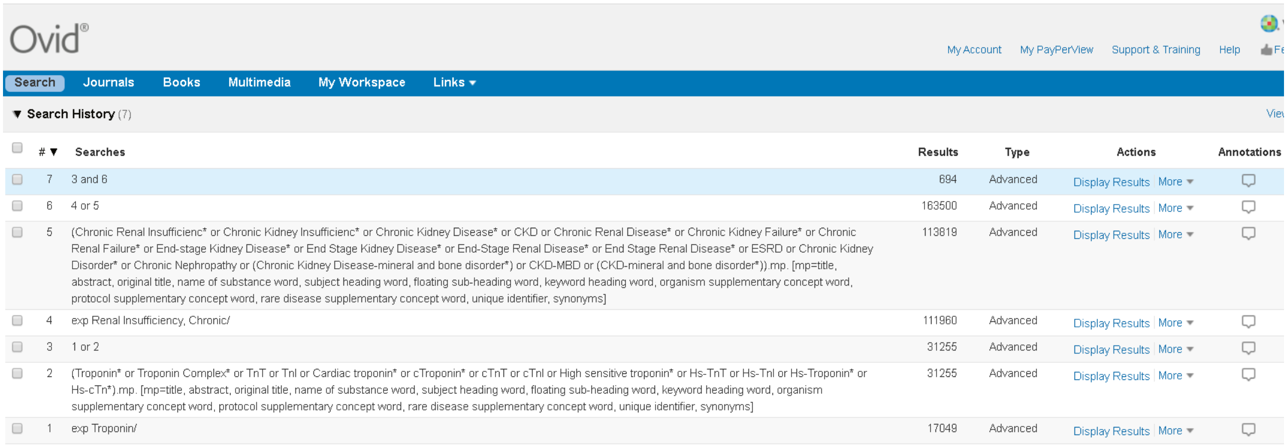

Ovid | My Account | My PayPerView | Support & Training | Help

Search | Journals | Books | Multimedia | My Workspace | Links

Search History (7)

#	Searches	Results	Type	Actions	Annotations
7	3 and 6	694	Advanced	Display Results More	
6	4 or 5	163500	Advanced	Display Results More	
5	(Chronic Renal Insufficienc* or Chronic Kidney Insufficienc* or Chronic Kidney Disease* or CKD or Chronic Renal Disease* or Chronic Kidney Failure* or Chronic Renal Failure* or End-stage Kidney Disease* or End Stage Kidney Disease* or End-Stage Renal Disease* or End Stage Renal Disease* or ESRD or Chronic Kidney Disorder* or Chronic Nephropathy or (Chronic Kidney Disease-mineral and bone disorder*) or CKD-MBD or (CKD-mineral and bone disorder*)).mp. [mp=title, abstract, original title, name of substance word, subject heading word, floating sub-heading word, keyword heading word, organism supplementary concept word, protocol supplementary concept word, rare disease supplementary concept word, unique identifier, synonyms]	113819	Advanced	Display Results More	
4	exp Renal Insufficiency, Chronic/	111960	Advanced	Display Results More	
3	1 or 2	31255	Advanced	Display Results More	
2	(Troponin* or Troponin Complex* or TnT or TnI or Cardiac troponin* or cTroponin* or cTnT or cTnI or High sensitive troponin* or Hs-TnT or Hs-TnI or Hs-Troponin* or Hs-cTn*).mp. [mp=title, abstract, original title, name of substance word, subject heading word, floating sub-heading word, keyword heading word, organism supplementary concept word, protocol supplementary concept word, rare disease supplementary concept word, unique identifier, synonyms]	31255	Advanced	Display Results More	
1	exp Troponin/	17049	Advanced	Display Results More	

Cochrane (21.01.2020, 197)

Exp Troponin/ OR (Troponin* OR Troponin Complex* OR TnT OR TnI OR Cardiac troponin* OR cTroponin* OR cTnT OR cTnI OR High sensitive troponin* OR Hs-TnT OR Hs-TnI OR Hs-Troponin* OR Hs-cTn*)

AND

Exp Chronic Renal Insufficiency/ OR (Chronic Renal Insufficienc* OR Chronic Kidney Insufficienc* OR Chronic Kidney Disease* OR CKD OR Chronic Renal Disease* OR Chronic Kidney Failure* OR Chronic Renal Failure* OR End-stage Kidney Disease* OR End Stage Kidney Disease* OR End-Stage Renal Disease* OR End Stage Renal Disease* OR ESRD OR Chronic Kidney Disorder* OR Chronic Nephropathy OR (Chronic Kidney Disease-mineral and bone disorder*) OR CKD-MBD OR (CKD-mineral and bone disorder*))

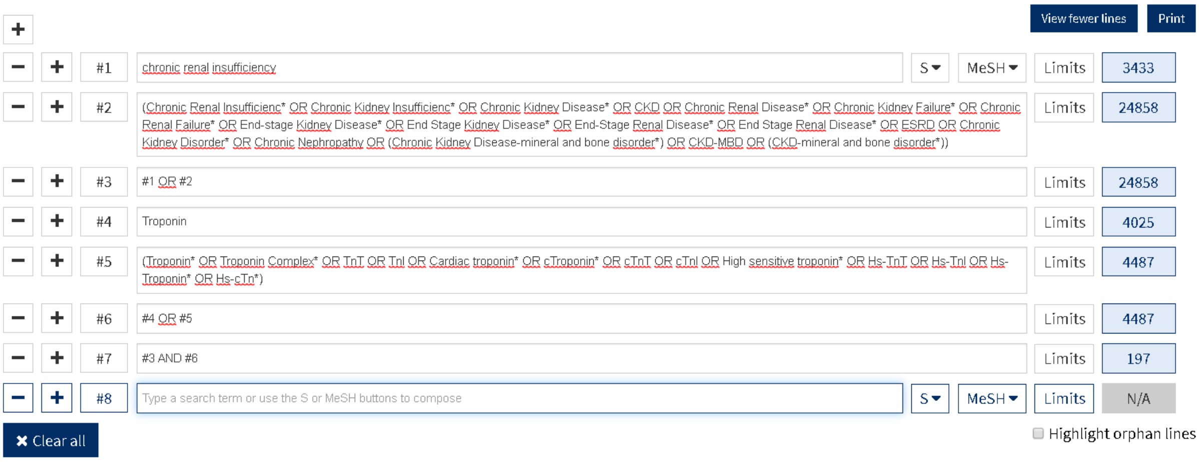

View fewer lines | Print

#	Search			Limits	Results
#1	chronic renal insufficiency	S	MeSH	Limits	3433
#2	(Chronic Renal Insufficienc* OR Chronic Kidney Insufficienc* OR Chronic Kidney Disease* OR CKD OR Chronic Renal Disease* OR Chronic Kidney Failure* OR Chronic Renal Failure* OR End-stage Kidney Disease* OR End Stage Kidney Disease* OR End-Stage Renal Disease* OR End Stage Renal Disease* OR ESRD OR Chronic Kidney Disorder* OR Chronic Nephropathy OR (Chronic Kidney Disease-mineral and bone disorder*) OR CKD-MBD OR (CKD-mineral and bone disorder*))			Limits	24858
#3	#1 OR #2			Limits	24858
#4	Troponin			Limits	4025
#5	(Troponin* OR Troponin Complex* OR TnT OR TnI OR Cardiac troponin* OR cTroponin* OR cTnT OR cTnI OR High sensitive troponin* OR Hs-TnT OR Hs-TnI OR Hs-Troponin* OR Hs-cTn*)			Limits	4487
#6	#4 OR #5			Limits	4487
#7	#3 AND #6			Limits	197
#8	Type a search term or use the S or MeSH buttons to compose	S	MeSH	Limits	N/A

Clear all | Highlight orphan lines

Web of Science (21.01.2020, 1036)

(**Troponin* OR Troponin Complex* OR TnT OR TnI OR Cardiac troponin*** OR cTroponin* OR cTnT OR cTnI OR High sensitive troponin* OR Hs-TnT OR Hs-TnI OR Hs-Troponin* OR Hs-cTn*)

AND

(**Chronic Renal Insufficienc* OR Chronic Kidney Insufficience*** OR Chronic Kidney Disease* OR CKD OR Chronic Renal Disease* OR Chronic Kidney Failure* OR Chronic Renal Failure* OR End-stage Kidney Disease* OR End Stage Kidney Disease* OR End-Stage Renal Disease* OR End Stage Renal Disease* OR ESRD OR Chronic Kidney Disorder* OR Chronic Nephropathy OR (Chronic Kidney Disease-mineral and bone disorder*) OR CKD-MBD OR (CKD-mineral and bone disorder*))

Search History:

Set	Results	Save History / Create Alert · Open Saved History	Edit Sets	Combine Sets ○ AND ○ OR · Combine	Delete Sets · Select All · ✕ Delete
# 3	1,036	#2 AND #1<br>*Indexes=SCI-EXPANDED, SSCI, A&HCI, CPCI-S, CPCI-SSH, ESCI Timespan=All years*	Edit	☐	☐
# 2	163,591	TS=(Chronic Renal Insufficienc* OR Chronic Kidney Insufficienc* OR Chronic Kidney Disease* OR CKD OR Chronic Renal Disease* OR Chronic Kidney Failure* OR Chronic Renal Failure* OR End-stage Kidney Disease* OR End Stage Kidney Disease* OR End-Stage Renal Disease* OR End Stage Renal Disease* OR ESRD OR Chronic Kidney Disorder* OR Chronic Nephropathy OR (Chronic Kidney Disease-mineral and bone disorder*) OR CKD-MBD OR (CKD-mineral and bone disorder*))<br>*Indexes=SCI-EXPANDED, SSCI, A&HCI, CPCI-S, CPCI-SSH, ESCI Timespan=All years*	Edit	☐	☐
# 1	43,893	TS=(Troponin* OR Troponin Complex* OR TnT OR TnI OR Cardiac troponin* OR cTroponin* OR cTnT OR cTnI OR High sensitive troponin* OR Hs-TnT OR Hs-TnI OR Hs-Troponin* OR Hs-cTn*)<br>*Indexes=SCI-EXPANDED, SSCI, A&HCI, CPCI-S, CPCI-SSH, ESCI Timespan=All years*	Edit	☐	☐
				○ AND ○ OR · Combine	Select All · ✕ Delete

PUBMED (21.01.2020, 753)

Troponin [MeSH Terms] OR (Troponin* OR Troponin Complex* OR TnT OR TnI OR Cardiac troponin* OR cTroponin* OR cTnT OR cTnI OR High sensitive troponin* OR Hs-TnT OR Hs-TnI OR Hs-Troponin* OR Hs-cTn*)

AND

Chronic Renal Insufficiency [MeSH Terms] OR (Chronic Renal Insufficienc* OR Chronic Kidney Insufficienc* OR Chronic Kidney Disease* OR CKD OR Chronic Renal Disease* OR Chronic Kidney Failure* OR Chronic Renal Failure* OR End-stage Kidney Disease* OR End Stage Kidney Disease* OR End-Stage Renal Disease* OR End Stage Renal Disease* OR ESRD OR Chronic Kidney Disorder* OR Chronic Nephropathy OR (Chronic Kidney Disease-mineral and bone disorder*) OR CKD-MBD OR (CKD-mineral and bone disorder*))

History

Download history Clear history

Search	Add to builder	Query	Items found	Time
#3	Add	Search **(#1 AND #2)**	743	11:04:06
#2	Add	Search **((Chronic renal insufficiency [MeSH Terms]) OR (Chronic Renal Insufficienc* OR Chronic Kidney Insufficienc* OR Chronic Kidney Disease* OR CKD OR Chronic Renal Disease* OR Chronic Kidney Failure* OR Chronic Renal Failure* OR End-stage Kidney Disease* OR End Stage Kidney Disease* OR End-Stage Renal Disease* OR End Stage Renal Disease* OR ESRD OR Chronic Kidney Disorder* OR Chronic Nephropathy OR (Chronic Kidney Disease-mineral and bone disorder*) OR CKD-MBD OR (CKD-mineral and bone disorder*)))**	196496	11:03:55
#1	Add	Search **((Troponin [MeSH Terms]) OR (Troponin* OR Troponin Complex* OR TnT OR TnI OR Cardiac troponin* OR cTroponin* OR cTnT OR cTnI OR High sensitive troponin* OR Hs-TnT OR Hs-TnI OR Hs-Troponin* OR Hs-cTn*))**	31673	11:03:19
